# The Long-Term Management of a Compound Odontoma: Successful Implant Rehabilitation After a Four-Year Follow-Up

**DOI:** 10.7759/cureus.82960

**Published:** 2025-04-25

**Authors:** Hulya Cerci Akcay, Eda Sir, Cagan Tas, Cagri Akcay, Oya Aktoren

**Affiliations:** 1 Pediatric Dentistry, Kocaeli Health and Technology University, Kocaeli, TUR; 2 Dentistry, Kocaeli Health and Technology University, Kocaeli, TUR; 3 Oral and Maxillofacial Surgery, Private Practice, Kocaeli, TUR; 4 Pediatric Dentistry, Istanbul University, Istanbul, TUR

**Keywords:** case report, compound odontoma, dental implant, impacted tooth, long-term follow-up, pediatric dentistry

## Abstract

Odontomas are the most prevalent odontogenic tumors; these are often asymptomatic and detected incidentally during routine imaging. Despite their benign nature, they can interfere with normal tooth eruption and require timely surgical management. We present a case of a 15-year-old patient with a compound odontoma associated with an unerupted maxillary canine, leading to delayed dental development. Following surgical excision, the impacted canine failed to erupt spontaneously, necessitating a long-term follow-up approach. After four years, implant placement was performed to restore function and esthetics successfully. This report highlights the importance of individualized treatment planning in odontoma cases, particularly when spontaneous eruption fails to occur, and emphasizes the role of implant rehabilitation in optimizing long-term oral health outcomes.

## Introduction

The term odontoma was first defined by Paul Broca in 1867 to describe any odontogenic tumor or tumor-like lesion of dental origin [1]. Odontomas are benign, hamartomatous developmental anomalies of dental tissues, arising from both epithelial and mesenchymal components [2]. They represent the most frequently diagnosed benign odontogenic tumors in routine radiographic examinations, observed in various age groups [3]. Odontomas are most commonly diagnosed within the first two decades of life, although cases have also been reported in later years [4]. Accounting for approximately 22% of odontogenic tumors, they are typically asymptomatic but may lead to bone expansion and impede the eruption of permanent teeth [4-6]. While often detected incidentally on radiographs, diagnosis can be challenging due to incomplete calcification. Their etiology remains uncertain, with proposed theories suggesting a multifactorial origin involving local, developmental, and genetic influences.

The World Health Organization (WHO) classifies odontomas into two types based on the degree of organization and differentiation of odontogenic tissues: compound odontomas (CpOD) and complex odontomas (CxOD) [7]. Compound odontomas, which exhibit an organized structure resembling normal dental tissue, are more frequently found in the anterior maxilla. In contrast, complex odontomas present as a disorganized radiopaque mass composed of calcified dental tissues and are predominantly located in the posterior mandible [3,8]. The differential diagnosis of odontomas includes ameloblastic fibro-odontoma, compound odontoma, and complex odontoma [5]. Compound odontomas are characterized by multiple tooth-like structures, whereas complex odontomas appear as irregular radiopaque masses. Ameloblastic fibro-odontomas typically show a mixed radiolucent-radiopaque appearance with notable soft tissue components. Accurate differentiation among these lesions relies heavily on detailed radiographic evaluation [6].

Compound odontomas constitute approximately 10% of odontogenic tumors, with a reported prevalence of 9-37%, while complex odontomas occur in 5-30% of cases [6]. In recent years, the use of advanced imaging modalities such as cone-beam CT (CBCT) has enhanced the diagnosis and surgical planning of odontomas, providing detailed three-dimensional visualization of the lesion. Surgical excision is the primary treatment to prevent recurrence, though complications such as eruption failure and bone defects may require further intervention. In such cases, implant-supported rehabilitation offers a viable functional and esthetic solution [4,9]. We discuss a case involving the surgical excision of an odontoma, followed by a comprehensive four-year postoperative assessment and subsequent implant-supported rehabilitation to restore function and esthetics. Although odontomas are well described in the literature, long-term outcomes following excision and implant rehabilitation in adolescent patients remain underreported. This report aims to contribute to addressing that gap.

## Case presentation

Initial findings

A 15-year-old male patient was referred to the dental clinic, reporting esthetic concerns related to the retained primary canine that had failed to exfoliate and appeared slightly discolored and malpositioned. The patient’s medical and family history was unremarkable, with no prior trauma or medication use, and his overall health status was reported as normal.

**Figure 1:**
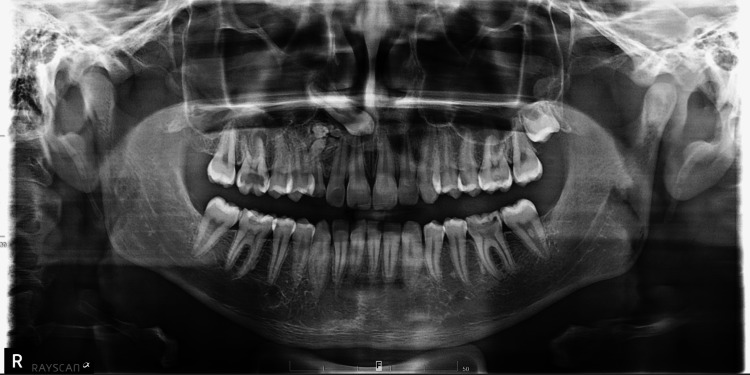
Panoramic radiograph showing radiopaque structures near the retained primary canine (53) and a superiorly displaced impacted maxillary canine

Clinical examination revealed a mixed dentition phase with a retained primary canine (tooth 53) that had failed to exfoliate. Extraoral assessment showed no facial asymmetry. Panoramic radiographic evaluation identified multiple tooth-like radiopaque structures in the apical region of the retained primary canine, accompanied by an impacted permanent maxillary canine displaced superiorly (Figure 1). The heterogeneous, likely multilocular, radiopaque mass was situated in the region between the upper right lateral incisor and the primary canine, extending distopalatally. The overlying mucosa appeared intact, with no evidence of erythema, ulceration, pain, or swelling.

Surgical procedure

Surgical excision remains the standard approach for odontoma removal, primarily due to its effectiveness in achieving complete lesion clearance and enabling histopathological evaluation. In this case, the decision to proceed with surgical excision was influenced by the lesion’s dense calcification and close proximity to critical anatomical structures, including the nasal floor. These anatomical challenges required direct visualization and precise manipulation, which are best achieved through conventional surgical access.

Although laser and piezoelectric surgery are considered minimally invasive alternatives for odontoma removal, their application in this case was limited. The dense calcified nature of the lesion and the risk of damaging adjacent structures could have led to incomplete removal or collateral tissue injury. Moreover, piezoelectric techniques may prolong operative time, and laser excision may lack the depth control required for safe and effective resection in such complex anatomical regions.

**Figure 2:**
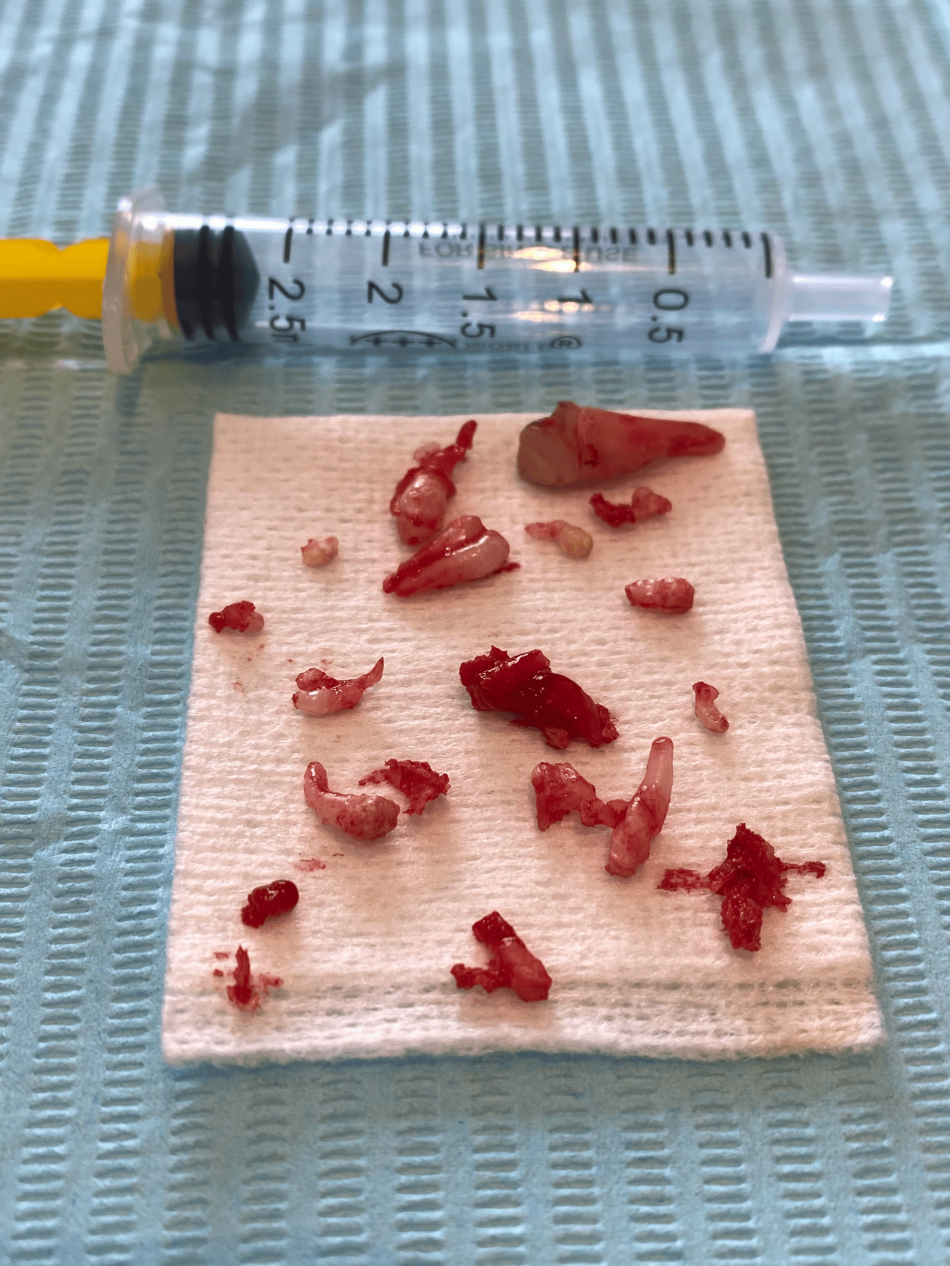
Surgically excised approximately 20 tooth-like structures of varying sizes and morphologies following the removal of a large calcified mass

In this case, a crestal incision with a distal releasing incision was performed in the alveolar region of tooth 53. A full-thickness mucoperiosteal flap was elevated to expose the surgical field. A large calcified mass was carefully extracted using a Bein elevator, with attention to preserving the adjacent permanent teeth. Approximately 20 tooth-like structures of varying sizes and morphologies were excised (Figure 2).

Postoperative period

**Figure 3:**
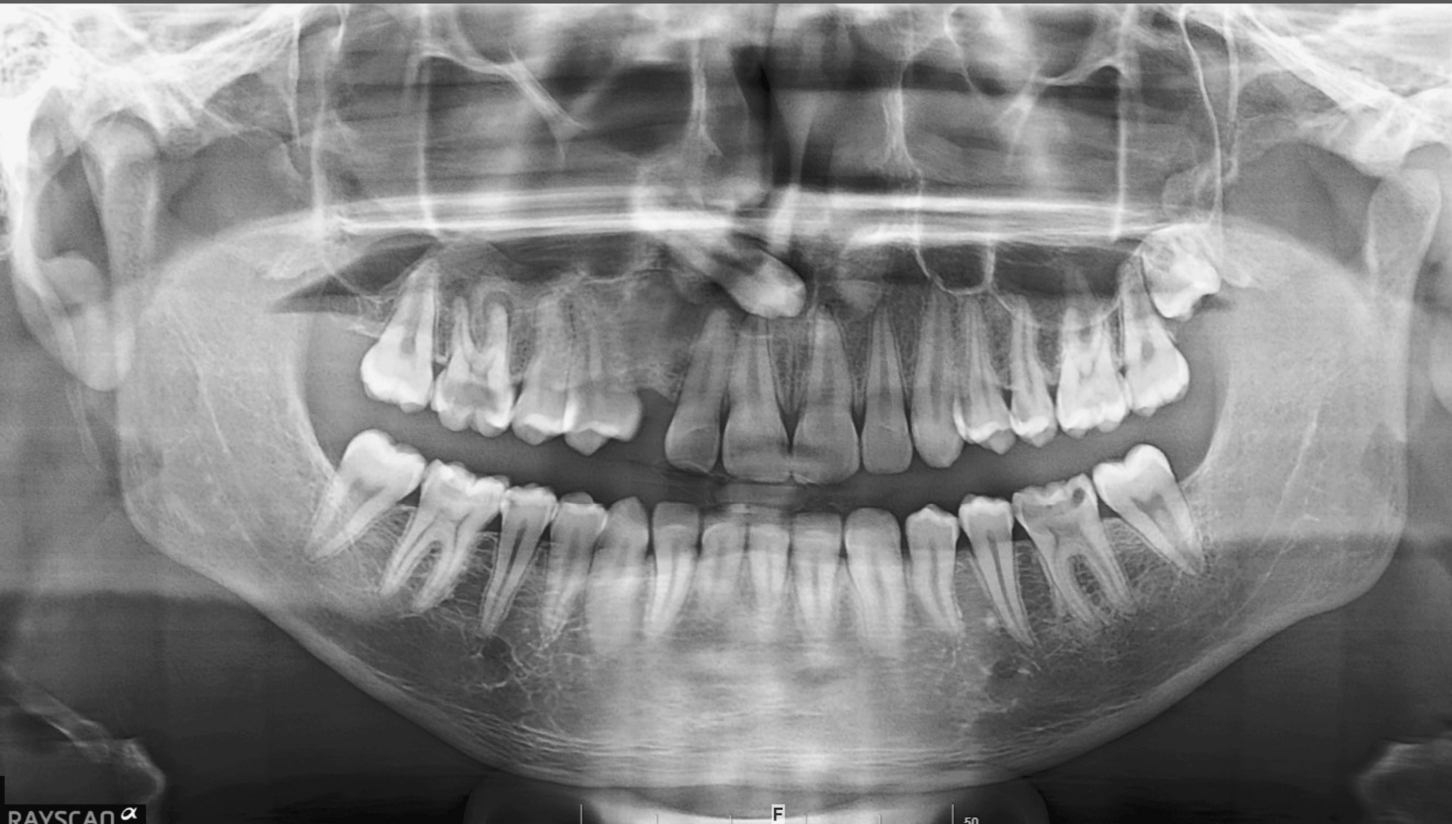
Postoperative panoramic radiograph confirming the complete removal of tooth-like structures following surgical excision

Intraoperative radiographic assessment confirmed the complete removal of all calcified components, including smaller fragments that had been radiographically obscured by larger masses (Figure 3). The surgical site was thoroughly irrigated, the flap was repositioned, and hemostasis was achieved before suturing with 3/0 silk. Postoperatively, the patient was prescribed antibiotics, anti-inflammatory agents, and analgesics, and was scheduled for regular follow-up visits to evaluate wound healing and monitor for spontaneous eruption.

Based on standard eruption timelines, the permanent maxillary canine typically erupts between the ages of 11 and 12 years [10]. In cases where odontomas are surgically removed before root formation is complete, spontaneous eruption is generally expected within 6-12 months. In the present case, however, no eruption was observed by the end of the first postoperative year. Clinical and radiographic assessments at that time confirmed the persistence of the impacted canine. Although a long-term follow-up protocol had been planned, the patient’s poor attendance disrupted continuity of care, and follow-up was conducted only intermittently over four years. Despite these limitations, progressive bone remodeling was observed at the surgical site.

Bone grafting, while frequently recommended to support bone preservation and facilitate tooth eruption, was not deemed necessary in this case. This decision was made based on clinical judgment, considering the patient’s age, limited socioeconomic resources, and inconsistent cooperation. Conventional orthodontic traction and periodontally accelerated osteogenic orthodontics (PAOO) were also evaluated, yet were considered clinically unfeasible due to the canine’s deep impaction, unfavorable angulation, and close proximity to the nasal floor. Ultimately, in light of the persistent edentulous space, failure of spontaneous eruption, and the patient’s functional and esthetic demands, implant-supported rehabilitation was selected as the definitive treatment approach.

Implant placement

**Figure 4:**
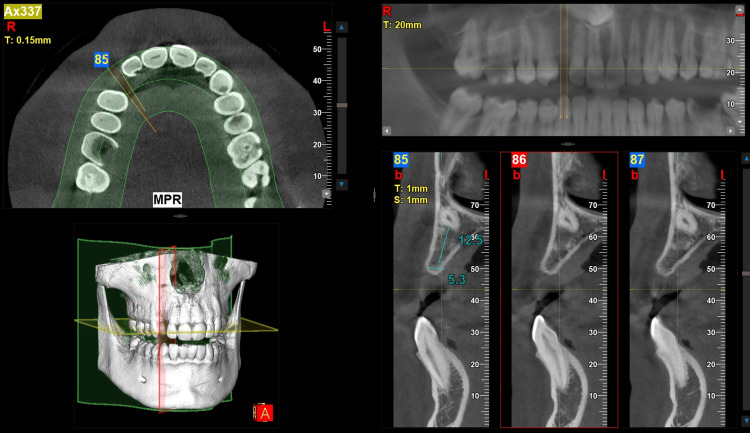
Cross-sectional CBCT image demonstrating adequate bone regeneration at the affected site following surgical excision Vertical and horizontal bone measurements were found to be 12.5 mm and 5.3 mm, respectively. Additionally, the impacted canine is observed in close proximity to the floor of the nasal cavity CBCT: cone-beam computed tomography

Following odontoma excision, the patient was monitored regularly over four years. At the 48th postoperative month, CBCT imaging confirmed adequate bone regeneration and sufficient bone volume for implant placement (Figure 4). Consequently, in June 2024, a 3.5 mm × 11 mm titanium dental implant was placed in the maxillary region under local anesthesia.

**Figure 5:**
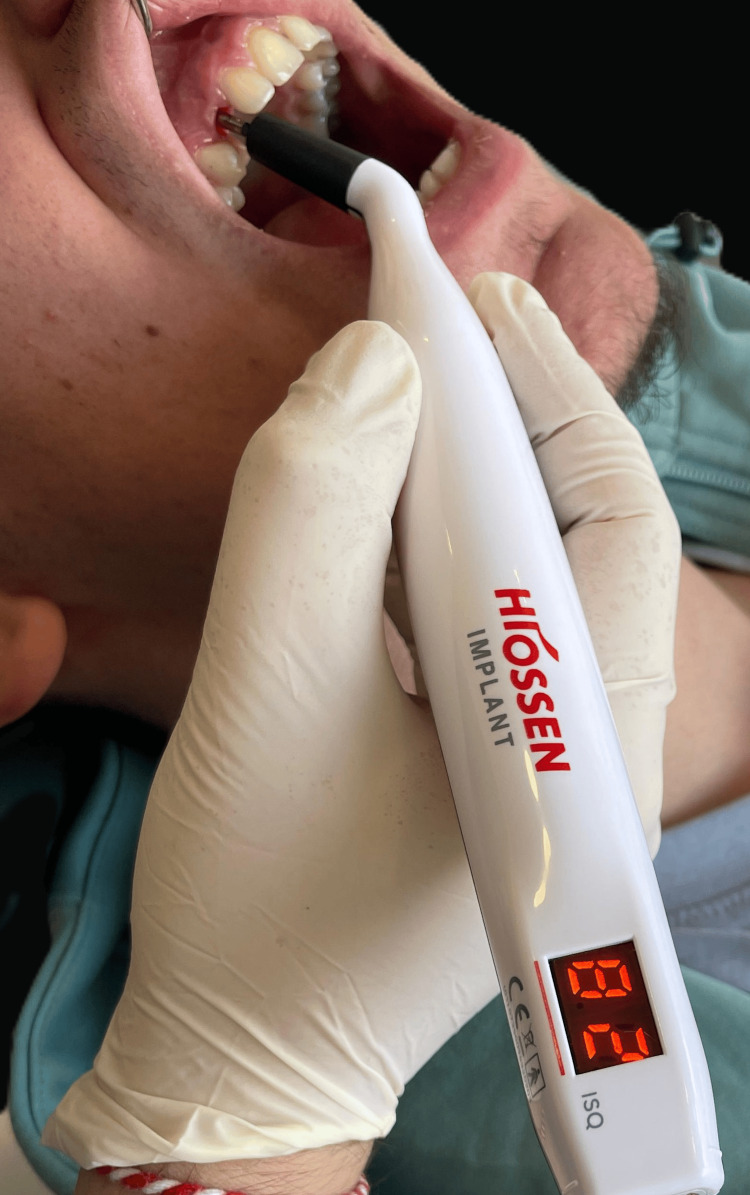
Assessment of implant stability using resonance frequency analysis Osseointegration was evaluated using an Osstell ISQ device, which measured an ISQ value of 82, indicating high primary stability suitable for prosthetic loading ISQ: implant stability quotient

**Figure 6:**
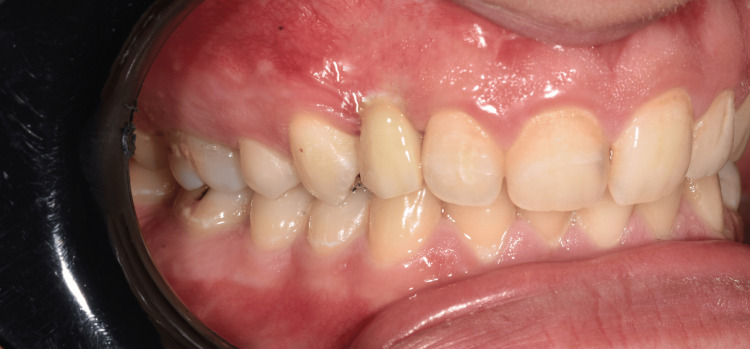
Intraoral view of monolithic zirconia implant-supported restoration Clinical photograph showing the final prosthetic outcome with a monolithic zirconia crown supported by a dental implant, demonstrating favorable gingival contour and integration with adjacent natural teeth

Three months post-placement, osseointegration was evaluated using resonance frequency analysis (RFA) with an Osstell device. The implant demonstrated a high implant stability quotient (ISQ) value of 82, indicating successful osseointegration and adequate stability for prosthetic loading (Figure 5). An ISQ value above 70 is widely accepted as a threshold for functional loading, indicating sufficient primary and biological stability [11]. Based on this assessment, prosthetic rehabilitation was initiated in September 2024, and a definitive monolithic zirconia crown was delivered within the same month (Figure 6). This timeline ensured optimal functional and esthetic restoration of the edentulous site.

Postoperative outcomes

**Figure 7:**
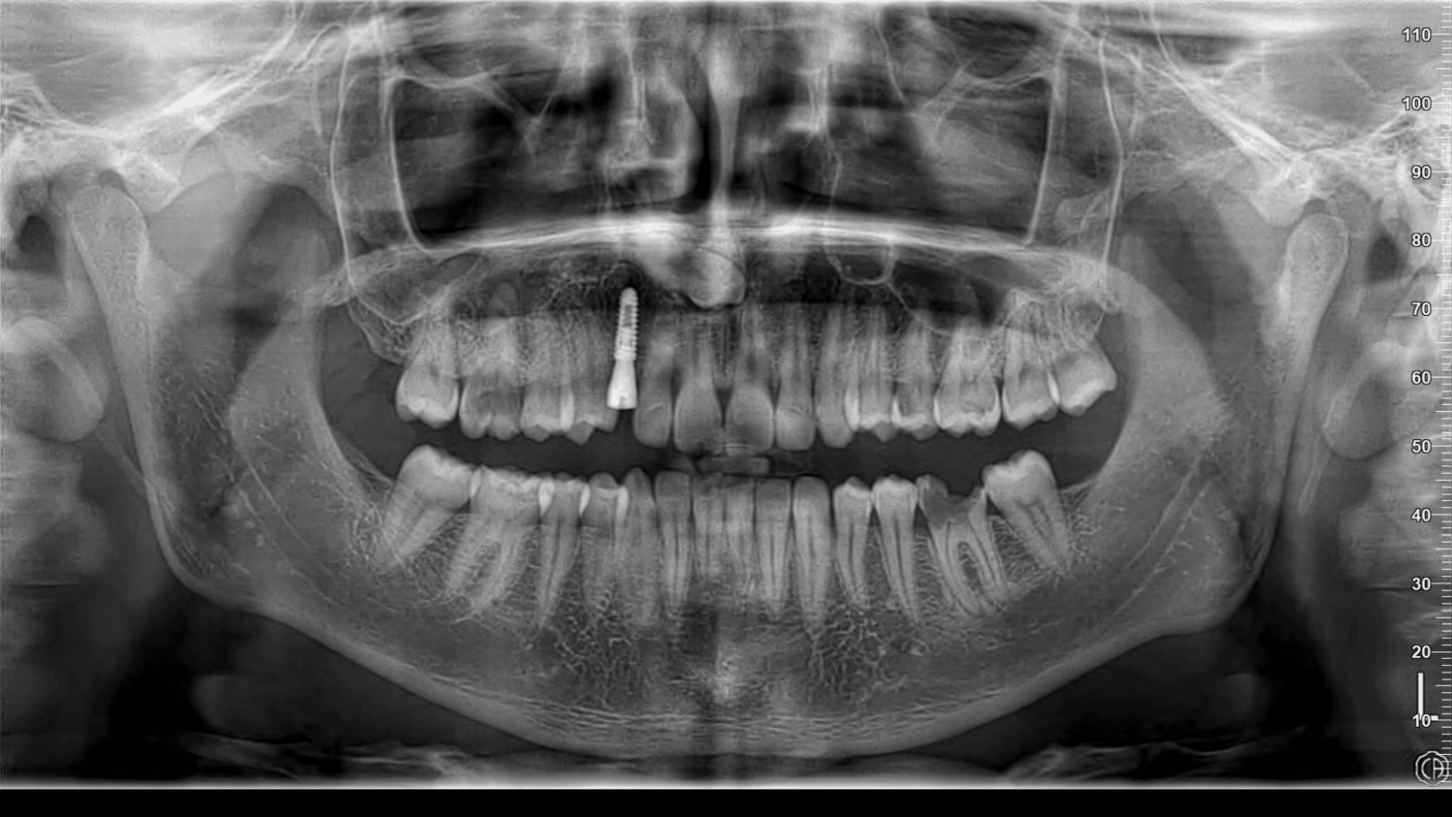
Follow-up radiographic assessments confirmed excellent osseointegration and stable bone levels

The patient experienced an uneventful recovery with no reported complications. Follow-up radiographic assessments confirmed excellent osseointegration and stable bone levels (Figure 7). Clinical evaluation demonstrated optimal functional and esthetic outcomes, indicating the success of implant rehabilitation.

Although radiographic examination revealed the presence of additional dental pathologies, no further interventions could be performed due to the patient's limited cooperation and failure to attend scheduled follow-up appointments consistently.

## Discussion

Odontomas are well-documented odontogenic tumors that frequently present as incidental findings during radiographic examinations [9]. Their structured composition, particularly in compound odontomas, distinguishes them from other developmental anomalies affecting tooth eruption [12]. In our case, the odontoma led to the impaction of a maxillary canine, a condition previously reported as a common sequela of such lesions [13]. Studies indicate that the presence of multiple small, tooth-like radiopaque structures is a hallmark of compound odontomas, often resulting in delayed or failed eruption of permanent teeth [14]. Our findings align with prior research demonstrating that spontaneous eruption following odontoma excision is unpredictable, emphasizing the necessity of long-term clinical and radiographic monitoring [6].

Early diagnosis and timely intervention are critical in managing odontomas to prevent secondary complications, such as root resorption or cystic transformation [15]. The present case underscores the importance of surgical excision as the primary treatment approach, in agreement with previous literature suggesting that complete removal reduces the risk of recurrence and facilitates future restorative planning [9]. While spontaneous eruption has been reported in some cases, it is not always a predictable outcome, necessitating additional interventions such as orthodontic traction or implant-supported rehabilitation [7]. The lack of spontaneous eruption in our patient, despite adequate bone healing, aligns with studies suggesting that delayed intervention and the prolonged presence of odontomas may impair the eruptive potential of impacted teeth [8].

The role of implant rehabilitation in odontoma-related edentulism remains an area of growing interest, particularly in pediatric and adolescent populations where active bone metabolism and ongoing skeletal growth influence both treatment timing and long-term outcomes [16]. Existing research has emphasized that alveolar bone volume must be carefully assessed before implant placement in young patients to ensure long-term success [17]. In this case, the absence of significant bone defects post-excision allowed for delayed implant placement without the need for bone grafting, a finding consistent with reports indicating that younger patients exhibit a higher potential for spontaneous bone regeneration [18]. A similar approach was reported by Dhawan et al. (2024), who achieved successful functional and esthetic outcomes following immediate implant placement with bone grafting in a case of labially impacted maxillary canine, reinforcing the feasibility of this protocol in select cases [19]. The successful osseointegration of the implant further reinforces the viability of this approach, providing both functional and esthetic benefits [20].

The localization of the odontoma in the maxillary anterior region, as observed in our case, is in accordance with epidemiological studies reporting a predilection for this site [21]. Compound odontomas are more frequently encountered in the anterior maxilla, whereas complex odontomas predominantly occur in the posterior mandible [3]. This distinction is crucial for accurate diagnosis and tailored management. Additionally, the observed high degree of morphodifferentiation within the excised odontoma mirrors findings from histopathological studies that have characterized these lesions as well-organized formations of dental tissues [22].

Ultimately, this case highlights the significance of individualized treatment planning in odontoma management. While early intervention may enhance the likelihood of spontaneous eruption, cases where eruption fails require alternative rehabilitative strategies [2,23]. The combination of surgical excision, long-term follow-up, and delayed implant placement ensured an optimal outcome for this patient. These findings reinforce the importance of a multidisciplinary approach, integrating surgical, orthodontic, and prosthetic expertise to achieve functional and esthetic success in odontoma cases.

## Conclusions

This case report underscores the importance of timely diagnosis, precise surgical management, and long-term follow-up in odontoma treatment. The successful excision of a compound odontoma, followed by implant rehabilitation, highlights implant placement as an effective solution for edentulism when spontaneous tooth eruption fails. Additionally, the case report suggests that pediatric patients may not require bone grafting post-excision due to their enhanced bone remodeling capacity. An interdisciplinary approach combining surgical, orthodontic, and prosthetic interventions is essential to achieving optimal functional and esthetic outcomes.
